# A Case Report of Wound-Vacuum-Assisted Closure (VAC) Treatment Following Pilonidal Cyst Excision

**DOI:** 10.7759/cureus.88193

**Published:** 2025-07-17

**Authors:** Jacob Ukleja, Vladimir Neychev

**Affiliations:** 1 General Surgery, University of Central Florida College of Medicine, Orlando, USA; 2 Surgery, University of Central Florida College of Medicine, Orlando, USA

**Keywords:** advanced wound care, general surg, pilonida cyst, postoperative wounds, wound care treatment

## Abstract

Pilonidal cysts are subcutaneous cysts that typically occur in the sacrococcygeal region and are more prevalent in obese, hirsute males. Pilonidal cysts are believed to be caused by a pore that forms as hairs become drawn deeper within the pore, ultimately creating a sinus. Different therapeutic options have been described, including excision with primary closure, healing by secondary intention, or flap creation; however, finding the optimal approach is a work in progress. We report a case of an otherwise healthy 28-year-old man who presented with a recurrent pilonidal cyst with a chronic sinus tract despite previous repeat incision and drainage procedures. The patient underwent pilonidal cyst excision with vacuum-assisted wound closure (wound-VAC) placement to facilitate the healing process. After two changes of the wound-VAC dressing (postoperative day eight), fresh granulations formed. The wound size and the wound depth decreased, requiring no further need for wound-VAC dressing. Over the next three weeks, the wound healed via secondary intention with a simple dry-to-dry gauze dressing with minimal intermittent pain and without any complications.

## Introduction

A pilonidal cyst is a subcutaneous cyst that typically occurs in the sacrococcygeal region within the cleft. Pilonidal cysts occur with a frequency of approximately 40 people per 100,000, with a higher incidence in males versus women [1]. Pilonidal cysts were originally believed to be the product of congenital skin abnormalities; however, the consensus theory now believes it is an acquired pathology caused by a pore that forms with hairs being drawn deeper within the pore, ultimately creating a sinus [2]. Common risk factors include obesity, local irritation, prolonged sedentary status, and increased hair density in the natal cleft region [3]. Patients may present with asymptomatic cavities or develop pain, inflammation, or acute infections worsened by movement or sedentary activity.

For chronic pilonidal disease, surgical excision is the standard treatment [4]. According to the American Society of Colon and Rectal Surgeons (ASCRS), treatment options often vary based on surgeon/patient preference [4]. Common options include incision and drainage, excision with primary closure (with midline or off-midline techniques), and excision with healing by secondary intention. Excision with healing by secondary intention has been found to be associated with lower recurrence rates compared to primary midline closure [4]. The average time to healing for a pilonidal cyst treated with surgical excision with secondary intention is approximately 46 to 55 days [5,6].

We present a case of a 28-year-old morbidly obese male patient presenting for excision of a pilonidal cyst treated with vacuum-assisted wound closure (wound-VAC) after excision to augment secondary intention healing.

## Case presentation

A 28-year-old Hispanic male presented to the clinic due to a three-year history of recurrent pilonidal cyst. He stated that the symptoms of pain fluctuate, ranging from minimal symptoms to severe pain aggravated with movement that is associated with feelings of fever and headache. He rated his current pain as a 5 out of 10, stating that the pain was exacerbated when pressure was applied to the area, such as when sitting. This cyst had been drained multiple times, but the symptoms continued to recur. He had noticed occasional clear fluid leaking from the site. The patient was referred by his primary care provider (PCP) for assessment of a pilonidal cyst for possible excision.

The patient denied chills, fatigue, fever, night sweats, or unintended weight changes. He denied chest pain, palpitations, lightheadedness, or syncope. He also denied cough, shortness of breath, or wheezing. He endorsed a small bump as well as localized pain and warmth in the sacrococcygeal region. He endorsed occasional non-bloody fluid leakage but denies any noticeable odor.

His medical history included dyslipidemia, for which he takes a rosuvastatin calcium 40 mg tablet orally once a day. His family history included high blood pressure and diabetes mellitus on both sides of the family. He was allergic to penicillin, latex, and iodine. The patient had femoral bone surgery 23 years prior. The patient denied smoking or recreational drug use. 

The patient’s temperature was 97.4 degrees Fahrenheit, blood pressure 130/85 mmHg, pulse 61 beats per minute, respiratory rate 16 breaths/minute on room air, and oxygen saturation of 98%. His height was 72 inches, his weight was 284 pounds, and his BMI was 38.51.

On initial physical examination, the patient was well-appearing, alert, and oriented, and in no acute distress. Lungs were clear to auscultation bilaterally, and heart function demonstrated a regular rate and sinus rhythm. A small, midline, sacrococcygeal dimple was observed with dark, coarse hair. The area was tender to palpation, and two small sinus openings were visible at the site without drainage.

Given the recurrence of the pilonidal cyst after multiple incisions and drainage, a decision was made to proceed with excision of the pilonidal cyst and chronic sinus tracts with wound-VAC placement to facilitate the postoperative wound healing. Following IV prophylactic antibiotics, the pilonidal cyst and sinus tracts were completely excised and dissected. After removal, the wound measured 4 cm long by 1.5 cm wide by 3 cm deep. The wound was irrigated with normal saline solution, and a black GranuFoam® (KCI USA, San Antonio, USA) foam portable wound-VAC kit was used as a wound dressing and placed at 125 mmHg of continuous suction. The patient was extubated and sent to the PACU in stable condition, where he was discharged the same day.

**Figure 1:**
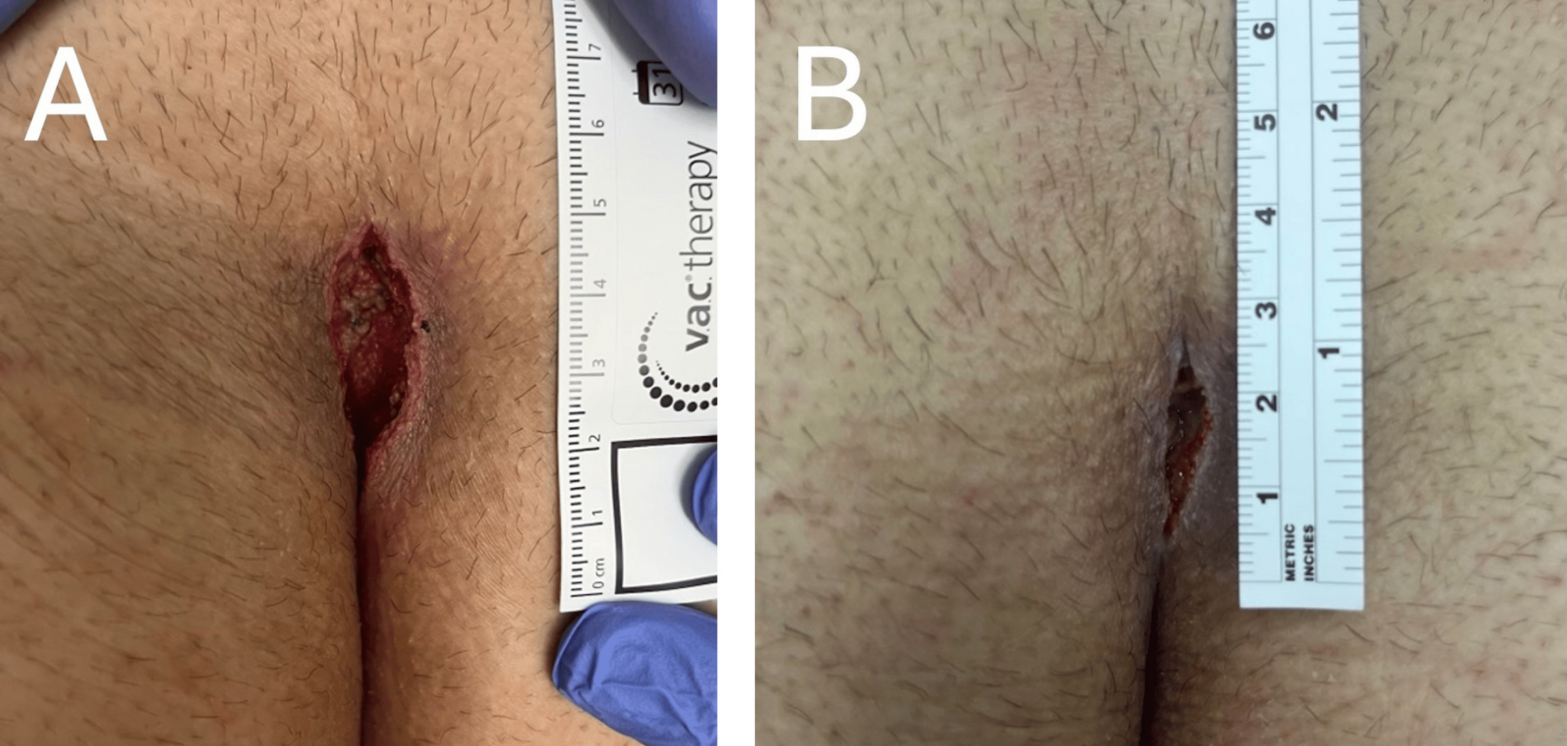
Postoperative pilonidal cyst wound healing A) Representative image of the healing pilonidal cyst wound three days postoperative; B) Representative image of the healing pilonidal cyst wound eight days postoperative.

The patient returned to the clinic on postoperative day three for wound-VAC dressing replacement. He complained of mild-to-moderate incisional pain that was managed well with oral medications. He denied fever, chills, fatigue, nausea, and vomiting. On physical examination, the wound site showed normal temperature, turgor, and softness. The wound was 3 cm long, 1.5 cm wide, and 2 cm deep with clean edges (Figure 1A). The wound-VAC dressing foam was downsized to 2.5 cm long, 1 cm wide, and 1.5 cm deep.

On postoperative day six, the patient’s wound continued to heal well and measured 3 cm long, 1.5 cm wide, and 1.5 cm deep with visible newly formed granulations. The wound-VAC foam was changed and again downsized to 2 cm long, 1 cm wide, and 1 cm deep. On postoperative day eight, the patient’s wound measured 2 cm by 0.5 cm by 1 cm (Figure 1B). No additional wound-VAC was deemed necessary at this time, and the patient was advised to continue cleaning the area and applying dry-to-dry gauze dressing once daily. At the one-month follow-up visit, the wound site showed virtually complete healing with excellent wound contracture. The patient was advised to return to the clinic as needed for any concerns.

## Discussion

Pilonidal cysts are subcutaneous, sacrococcygeal cysts that can present asymptomatically or progress until they ultimately become infected, resulting in abscesses or chronic draining sinuses. Pilonidal cysts are diagnosed clinically based on the presence of characteristic midline pits in the gluteal cleft and symptoms.

There are multiple treatment options, including incision and drainage, excision with primary closure (with midline or off-midline techniques), advancement flaps, rotational flaps, and excision with healing by secondary intention [4,7]. Incision and drainage are a comparatively simple and quick procedure that can be performed under local anesthesia, providing immediate relief from pain and infection with no wound formation [8]. The recurrence rate of pilonidal cysts treated with incision and drainage can range between 15% and 40%, often necessitating additional treatment [4]. Excision with primary closure is another treatment modality that has been found to have a recurrence rate of 8.7% and a healing time of 23-65 days [4]. Compared to off-midline closures, midline closure wounds were found to take 5.4 days longer to heal and had a higher rate of infection (relative risk 4.70, 95% confidence interval 1.93 to 11.45) [9]. The modified Limberg flap is an advancement flap option where, following cyst excision, a rhomboid-shaped defect is produced and subsequently closed with a transposition flap. 

Although this approach was found to have a recurrence rate of 7.4%, the larger area of tissue mobilization and increased rate of wound dehiscence require ongoing wound care time for complete healing [7]. The rotational gluteal flap approach involves rotating the flap from the gluteal region to cover the excision defect. It has been shown to have recurrence rates of 3.4%, a lower complication rate, and a better cosmetic appearance when compared to a modified Limberg flap [7]. Excision with open wound healing by secondary intention has been found to close at a slower rate of 46 to 55 days and requires more intensive wound care [5,6]. Wound healing by secondary intention presents with a recurrence rate of 5.3% [4].

A wound-VAC utilizes controlled suction to remove excess fluid and debris from a wound. The wound-VAC device works by applying controlled negative pressure, typically set at 125 mmHg, to the wound bed through a sealed system. This is transmitted via an open-cell foam dressing placed within the wound cavity. The device promotes secondary intention wound healing through mechanisms including increased blood flow, enhanced granulation tissue formation, and reduction of edema [10-12]. The rationale for supplementing the secondary intention healing with wound-VAC was to expedite recovery and minimize infection.

Currently, the addition of wound-VAC treatment is not considered one of the standard treatments based on the 2019 ASCRS guidelines [4]. There are a few small-sized studies discussing the implementation of wound-VAC treatment to further decrease healing time. In a study with 19 patients, Banasiewicz et al. found that implementing wound-VAC therapy significantly decreased the time of wound healing, absenteeism from work, and postoperative late pain [13]. In another study of 49 patients, Biter et al. found a positive effect on wound size reduction in the first two weeks post-surgical incision with wound-VAC but no significant difference between wound healing resolution and time to resume daily life activities [14].

This patient had multiple recurrences of pilonidal cysts following incision and drainage and, therefore, was amenable to surgical excision of the cyst with wound-VAC placement. Within eight days postoperatively, this patient’s wound was healed by secondary intention to the point of no longer needing wound-VAC with no symptoms other than mild pain consistent with any excision treatment. The patient did comment on how his insurance was not originally covering the wound-VAC component, an issue that was in the process of remediation. Insurance companies may not cover wound-VAC treatment for pilonidal cyst excision due to the limited evidence discussing the benefits of expediting healing. Another limitation is that not all healthcare facilities may have access to wound-VAC devices, especially in rural or underserved areas. Trained healthcare professionals are also needed to monitor and adjust the device.

## Conclusions

Pilonidal cysts are subcutaneous, sacrococcygeal cysts that can present asymptomatically or progress until they ultimately become infected and painful, drastically affecting routine activities of daily living. This report highlights a common presentation of a recurrent pilonidal cyst in an obese, hirsute 28-year-old male treated with surgical excision with non-traditional wound-VAC closure to improve secondary intention healing. In smaller studies, wound-VAC treatment has been found to promote faster wound size reduction as well as decrease the time of wound healing, absenteeism from work, and postoperative late pain.

Further larger-scale randomized clinical studies will be needed to study the advantages of wound-VAC healing as a critical additional modality for pilonidal cyst treatment.
